# Reproductive medical providers’ behaviors, considerations, and plans for fertility treatments during the COVID‐19 pandemic in Japan: A nationwide web‐based survey

**DOI:** 10.1002/rmb2.12372

**Published:** 2021-03-22

**Authors:** Kuniaki Ota, Osamu Tsutsumi, Tasuku Mitani, Yoshiharu Morimoto, Atsushi Tanaka, Yutaka Osuga, Toshifumi Takahashi, Yoshihiko Hosoi

**Affiliations:** ^1^ Department of Obstetrics and Gynecology Toho University Tokyo Japan; ^2^ Center for Human Reproduction and Gynecologic Endoscopy Sanno Hospital Tokyo Japan; ^3^ Graduate School of Biology‐Oriented Science and Technology Kindai University Wakayama Japan; ^4^ HORAC Grand Front Osaka Clinic Osaka Japan; ^5^ Saint Mother Obstetrics and Gynecology Clinic Institute for ART Fukuoka Japan; ^6^ Department of Obstetrics and Gynecology The University of Tokyo Tokyo Japan; ^7^ Fukushima Medical Center for Children and Women Fukushima Medical University Fukushima Japan

**Keywords:** nationwide survey, oocyte retrieval, pregnancy, questionnaires, severe acute respiratory syndrome

## Abstract

**Purpose:**

This study was conducted to investigate how the COVID‐19 pandemic has impacted reproductive medical providers’ behaviors and considerations, including their concerns regarding the necessity of fertility treatments.

**Methods:**

A web‐based questionnaire was distributed to Japan Society of Fertilization and Implantation (JSFI) members from May 18 through May 31, 2020 to survey their professional behaviors and concerns during the COVID‐19 pandemic.

**Results:**

Most survey participants reported a decrease in the number of patients and a decrease in their workload. Most also believe that the use of fertility treatments will return to the pre‐pandemic levels after the COVID‐19 pandemic ends. Additionally, more than half of the participants reported that they consider fertility treatment neither necessary nor unnecessary during the COVID‐19 pandemic.

**Conclusions:**

At the institute where reproductive medical providers worked in Japan, the number of outpatients and the working time tended to decrease during the COVID‐19 pandemic. However, amid fears of infection during the COVID‐19 pandemic, the reproductive medical providers working at fertility institutes in Japan have remained engaged in their work with a sense of mission and hope.

## 1 INTRODUCTION

The Coronavirus Disease 2019 (COVID‐19) epidemic emerged in Wuhan, China, spreading nationwide and then to several other countries between December 2019 and early 2020. Unfortunately, the disease has since spread globally. On March 11, 2020, the World Health Organization (WHO) declared COVID‐19 a pandemic,^1^ and the Japanese government declared a state of emergency to change the course of the outbreak.

The COVID‐19 pandemic suddenly altered health care services, including reproductive medical care, necessitating the reorganization of workplaces and work practices worldwide. At the time of this writing, elective procedures and treatments have been postponed or canceled, in line with the recommendations of health authorities and associations, including the CDC, the WHO, the American Society for Reproductive Medicine (ASRM), the European Society for Human Reproduction and Embryology (ESRHE), RCOG, ACOG, and the Japan Society for Reproductive Medicine (JSRM). In particular, most fertility treatments with the exception of fertility preservation for cancer patients were suspended.

The outbreak of COVID‐19 in the first few months of 2020 suddenly and unexpectedly confronted fertility patients with a new set of losses and uncertainties. Owing to the implementation of stringent social distancing measures and the suspension of “non‐essential” medical treatments and procedures, fertility treatments were halted in the US, the UK, and other European countries.^2^ Fertility treatments may have decreased because of the COVID‐19 pandemic even though vast numbers of assisted reproductive technology (ART) cycles have been reported in Japan in recent years.^3^

Although there are no available data indicating whether the number of fertility treatments has been affected by the COVID‐19 pandemic, previous studies have reported that reproductive medical providers faced many problems during the 2003 SARS outbreak.^4^, ^5^, ^6^, ^7^ Indeed, studies have revealed that reproductive medical providers feared contagion and were concerned about the possibility of infecting their families, friends, and colleagues.^4^ They also reported feeling stigmatized ^4^, ^5^ and were reluctant to work; some providers even contemplated resignation.^5^ Reports of the SARS outbreak also describe reproductive medical providers experiencing high levels of stress and anxiety.^6^ Similarly, health care professionals treating and caring for patients with infertility may be experiencing similar behaviors and mental health issues due to the COVID‐19 pandemic.

The primary purpose of this study was to conduct a nationwide survey to investigate reproductive medical providers’ work‐related actions and considerations during the COVID‐19 pandemic. Additionally, we aimed to clarify their perceptions about whether fertility treatment was necessary during the COVID‐19 pandemic based on discussions about the social value of offering fertility treatments and on whether it is justifiable to suspend them in the event of a health crisis of this proportion.

## 2 MATERIALS AND METHODS

We conducted a web‐based survey about behaviors and responses to the tumultuous COVID‐19 pandemic situation among the members of the Japan Society of Fertilization and Implantation (JSFI). A self‐report questionnaire was initially designed to survey behaviors and responses during the COVID‐19 pandemic (Table 1). The questionnaire consisted of 17 multiple‐choice questions; for each item, one or more answers could be chosen. If a participant's answer did not match any of the provided choices for an item, the participant was permitted to provide their own response. Six of the 17 items collected demographic information (ie, age, gender, and job title) and information about the fertility institute (ie, prefecture and the number of oocyte retrievals). The other 11 items involved participants’ behaviors and concerns related to ART activity in their institute during the COVID‐19 pandemic. Information about this survey was available on the JSFI website, and surveys were simultaneously emailed to 1641 JSFI members who were registered on the mailing list of the JSFI on May 18, 2020.^7^ The survey website contained a statement explaining to the participants that responding to the survey implied that the participants had provided informed consent. Participants were able to answer the survey from May 18, 2020, to May 31, 2020.

**TABLE 1:** The Questionnaire of behaviors, considerations, and plans in reproductive medical providers

Q1. How old are you?
Q2. What is your gender? 1. Male 2. Female
Q3. What is your job title? 1. Medical doctor 2. Nurse 3. Embryologist 4. Counselor 5. Medical clerk 6. None of the above
Q4. What prefecture do you live in?
Q5. Are you currently working in a fertility institute? 1. Yes 2. No
Q6. How many oocyte retrievals have been performed at the institute you belong to?
Q7. What are your thoughts as a medical provider on the COVID‐19 pandemic? 1. I want to leave the medical field because of fear of nosocomial infection. 2. I am not going to leave the medical field, although I am afraid. 3. I am not exposed to the danger of nosocomial infection because I am very careful. 4. No association of nosocomial infection with me 5. None of the above
Q8. What preventive methods for nosocomial infection are being employed in you institute? 1. Frequent hand washing and sanitizing 2. Wearing protective clothing, protective eyewear, and a face mask 3. Keeping recommended physical distance 4. Improving the ventilation in the medical facilities 5. Staying in a hotel by myself to avoid intra‐familial infection 6. None of the above
Q9. What work‐related change have you experienced as a reproductive medical provider compared to before the COVID‐19 pandemic? 1. No change compared to before the COVID‐19 pandemic 2. Busy compared to before the COVID‐19 pandemic 3. Not busy compared to before the COVID‐19 pandemic 4. Decreased working days 5. Fired 6. Quitting by myself 7. None of the above
Q10. Do you think fertility treatment is necessary during the COVID‐19 pandemic? 1. Yes 2. No 3. Not sure
Q11. Do you explain the association of COVID‐19 with fertility treatment to the patients undergoing fertility treatment? 1. Yes 2. No 3. Explained, if the patient asked 4. None of the above
Q12. What materials do you use when you explain the association of COVID‐19 with fertility treatment to the patient? 1. Statement of JSRM 2. Joint statement of JSOG, JAOG, and JSIDOG 3. Statement of JISART 4. Statements from foreign societies of other countries 5. Statement from the Ministry of Health, Labour and Welfare 6. Others JSRM: Japan Society for Reproductive Medicine, JSOG: Japan Society for Obstetrics and Gynecology, JAOG: Japan Association for Obstetrics and Gynecology, JSIDOG: Japan Society for Infectious Diseases in Obstetrics and Gynecology, JISART: Japanese Institution for Standardizing Assisted Reproductive Technology
Q13. What is the typical response of a patient who received an explanation about the association of COVID‐19 with fertility treatment by reproductive medical providers? 1. Eager to continue fertility treatment with the consent of the couple (including natural and IUI) 2. Eager to undergo only oocyte retrieval and freeze all blastocysts afterward with the consent of the couple 3. Eager to postpone all fertility treatments with the consent of the patient 4. Eager to continue fertility treatment except IVF with the consent of the patient 5. Eager to decide after consultation with family 6. None of the above 7. Others Q14. Do you feel that the number of patients has decreased since the COVID‐19 pandemic began? 1. Yes 2. No 3. Neither
Q15. Which of the following aspects have you had anxieties about since the COVID‐19 pandemic began? 1. Health and mental status 2. Income 3. Employment 4. Medical techniques and knowledge 5. Family 6. The patient`s fertility treatment 7. Others
Q16. Would you hope to keep working as a reproductive medical provider after the end of the COVID‐19 pandemic? 1. Yes 2. Transfer to other medicine‐related occupations 3. Transfer to other occupations 4. None of the above
Q17. What do you think will happen to fertility treatments after the end of the COVID‐19 pandemic? 1. Expected to increase after the end of the COVID‐19 pandemic 2. Expected to not change, although the number of patients may temporarily increase after the end of the COVID‐19 pandemic 3. Expected to not change after the end of the COVID‐19 pandemic 4. Expect no decrease in the number of patients for fertility treatment, although the number of ART including IVF may decrease after the end of the COVID‐19 pandemic 5. Expect minor (20%‐30%) decrease in the number of patients for fertility treatment after the end of the COVID‐19 pandemic 6. Expect major (more than 50%) decrease in the number of patients for fertility treatment 7. Others

## 3 RESULTS

**FIGURE 1:**
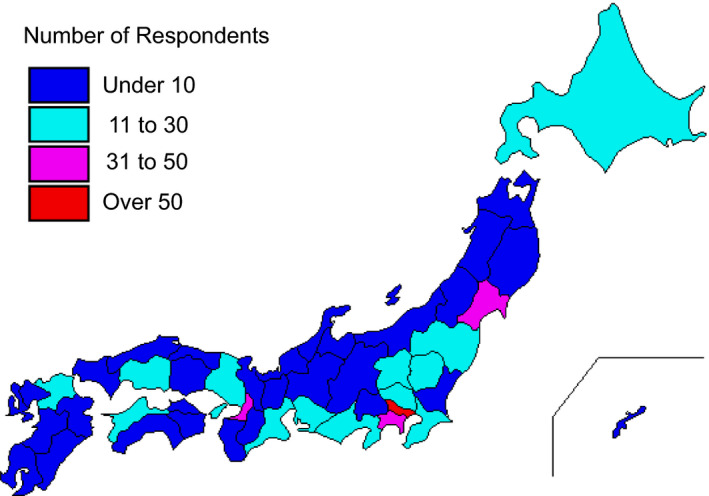
Cartographic representation of prefecture level information for the distribution of participants in this survey of reproductive medical providers in Japan regarding COVID‐19

**FIGURE 2:**
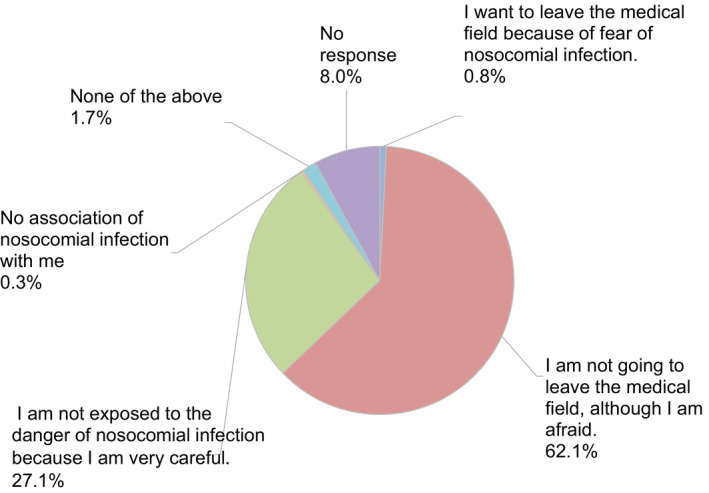
Reproductive medical provider's thoughts on remaining active in the field (or leaving) during the COVID‐19 pandemic

**FIGURE 3:**
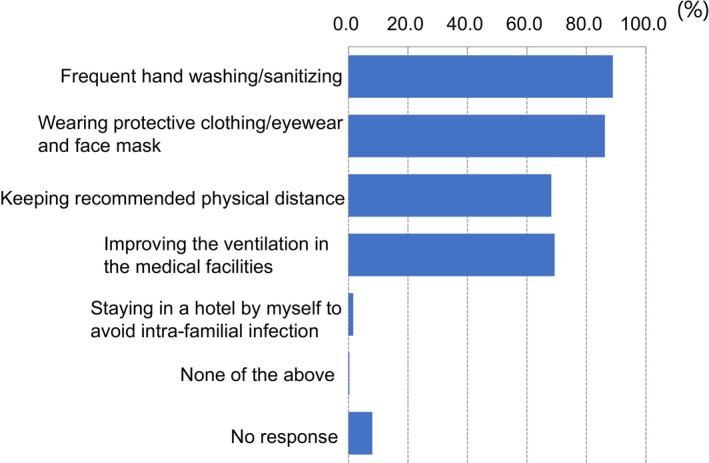
The preventive methods used by fertility/reproductive medical providers to avoid nosocomial infection in each institute

A total of 638 valid completed surveys (response rate of 44.2%) were obtained from 1641 members (Table 2) across every prefecture in Japan. Among the participants who completed the entire survey, 50.3% were women and 49.7% were men. More participants were in their 40s than any other age group; however, we received valid responses from all age groups, with participants’ ages ranging from 29 years to over 70 years. About 48.9% of the respondents were medical doctors and 40.8% were embryologists, even though embryology is the main aspect of the survey. Participants who work at high volume fertility centers—centers reporting more than 1000 cycles of oocyte retrievals—made up 17.2% of all respondents. More than 80% of participants were from urban areas (Figure 1). The majority of participants (89.2%) indicated they were not willing to abstain from working in the field because of the pandemic, although most of them feared nosocomial COVID‐19 infection (Figure 2). Most participants (86.2%) reported practicing disinfection behaviors (88.9%) and using protective equipment (86.2%) most of the time when working directly with patients (Figure 3).

**TABLE 2:** Characteristics of reproductive medical provider participants and the fertility institute volume of oocyte retrievals

	n	%
Age (years)		
Under 29	57	8.9
30s	151	23.7
40s	206	32.3
50s	131	20.5
60s	80	12.5
Over 70	13	2.0
Total	638	
Gender		
Male	321	50.3
Female	317	49.7
Total	638	
Occupation		
Medical doctor	312	48.9
Nurses	24	3.8
Embryologist	260	440.8
Psychologist	6	0.9
Officer	8	1.3
Others	28	4.4
Total	638	
Number of oocyte retrievals		
Under 100	75	11.8
101 to 300	174	27.3
301 to 600	121	19.0
601 to 800	50	7.8
801 to 1000	54	8.5
Over 1000	110	17.2
None	54	8.5

**FIGURE 4:**
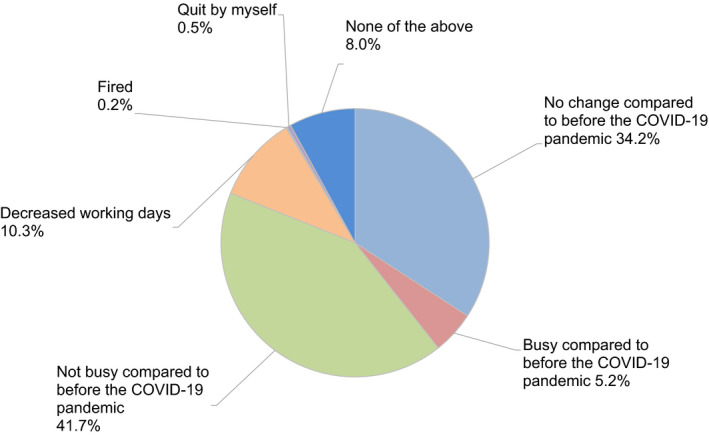
The working status of reproductive medical providers during the COVID‐19 pandemic

**FIGURE 5:**
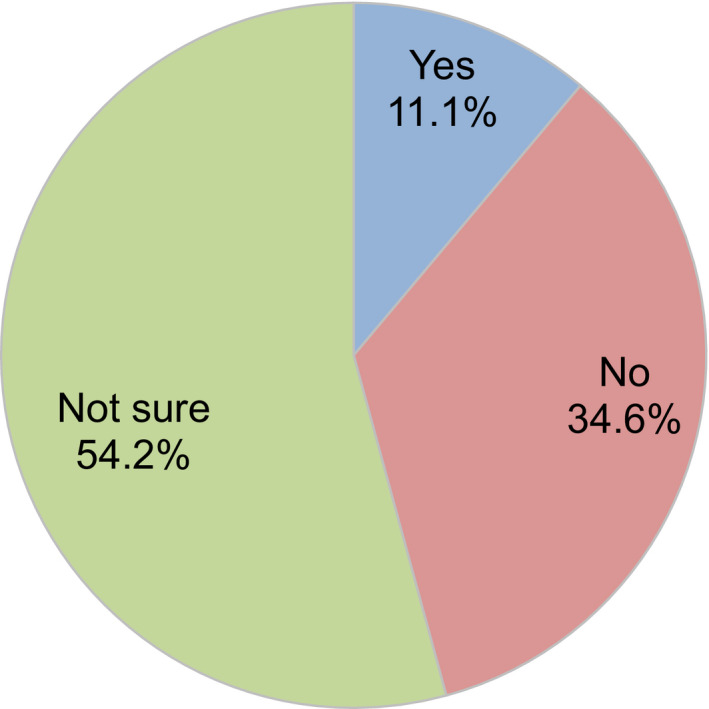
Opinions of fertility treatment/reproductive medical providers regarding whether fertility treatment during the COVID‐19 pandemic is necessary or unnecessary

**FIGURE 6:**
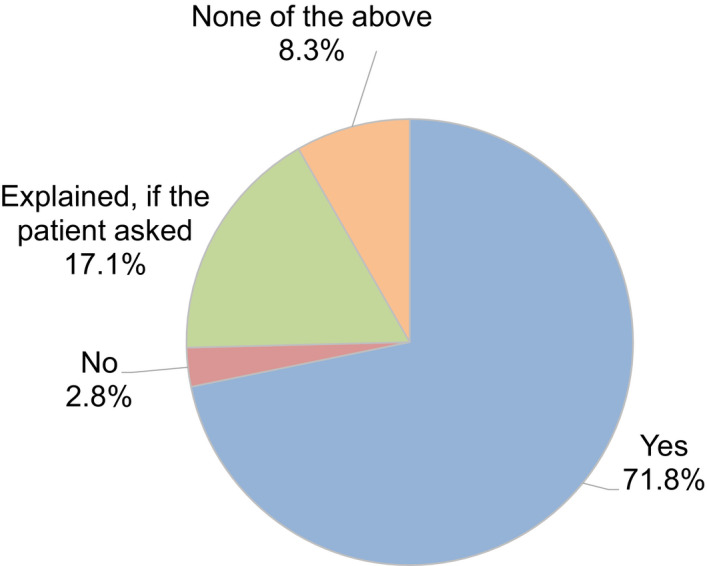
The status of fertility providers having provided an explanation to their patients regarding the association of COVID‐19 and fertility treatment

**FIGURE 7:**
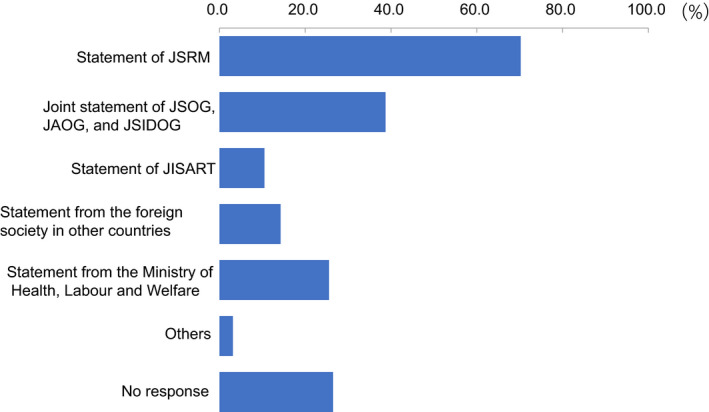
What materials do you use when you explain the association of COVID‐19 with fertility treatment to the patient?

Regarding levels of ART activity during the COVID‐19 pandemic, 34.2% of participants indicated there had been no change compared to before the pandemic, but 41.7% indicated reductions in ART activity at their clinic (Figure 4). Approximately one‐third (34.6%) of participants consider it necessary to continue fertility treatment during the COVID‐19 pandemic, while 50% said it was neither necessary nor unnecessary (Figure 5). When asked if they explained to patients how COVID‐19 might impact their fertility treatments, 71.8% of participants said that they had this discussion (Figure 6); 70.2% explained this aspect using the JSRM guidance statement on fertility care during the COVID‐19 pandemic. Although participants included more members of the JSOG than JSRM, the reported use of their joint statement was much lower than the reported use of the statement from JSRM (Figure 7).

**FIGURE 8:**
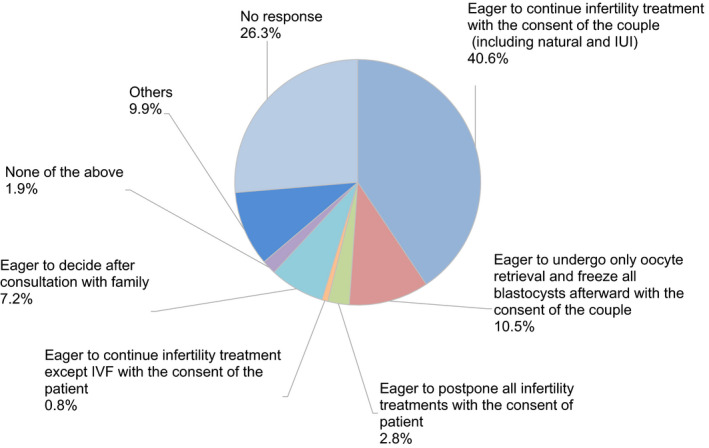
The patient`s response after explaining the association of the COVID‐19 and fertility treatment

**FIGURE 9:**
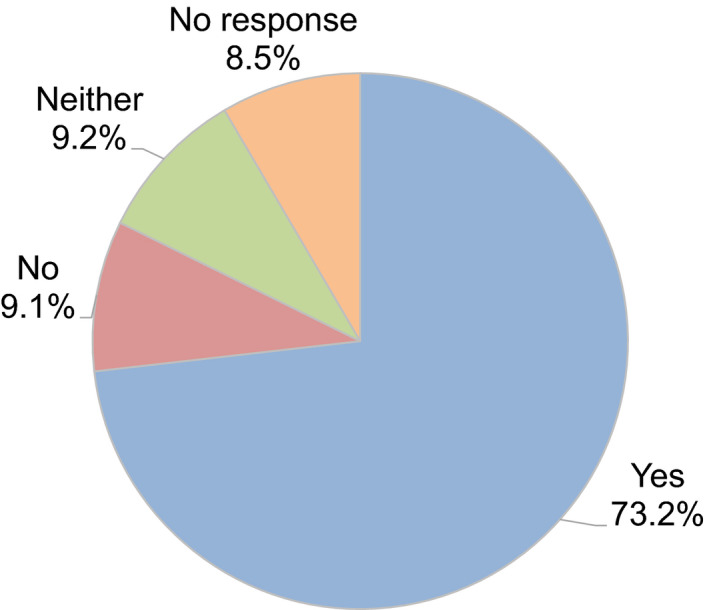
Have you seen a decrease in outpaitents since the start of the pandemic? Yes or No?

Regarding the decision as to whether to continue fertility treatment during the COVID‐19 pandemic, 40.6% of couples decided to continue fertility treatment. In contrast, 10.5% of couples hesitated to undergo embryo transfer because of the advice of the guidance statements, such as the statement from the JSRM, although they underwent oocyte retrieval following controlled ovarian stimulation (Figure 8). A decrease in outpatient fertility treatment due to the pandemic was reported by 73.2% of participants (Figure 9). Anxiety about self‐status and family regarding the disease was indicated by 49.2% and 32.0% of participants, respectively, although 31.3% of participants anxiously considered whether the patient`s fertility treatment could be continued or not.

**FIGURE 10:**
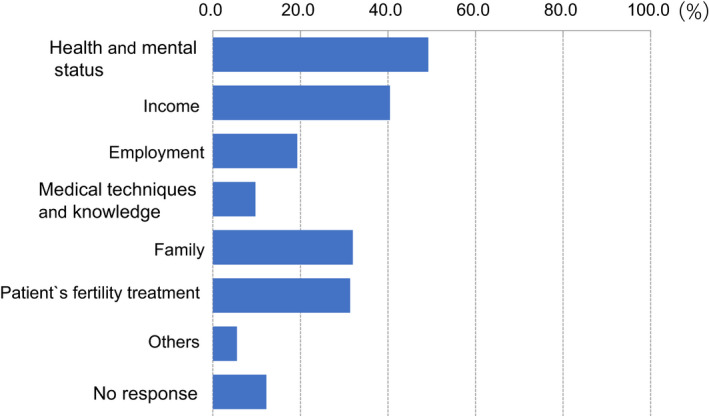
The distribution of issues causing concern and anxiety for reproductive medical professionals during the COVID‐19 pandemic

**FIGURE 11:**
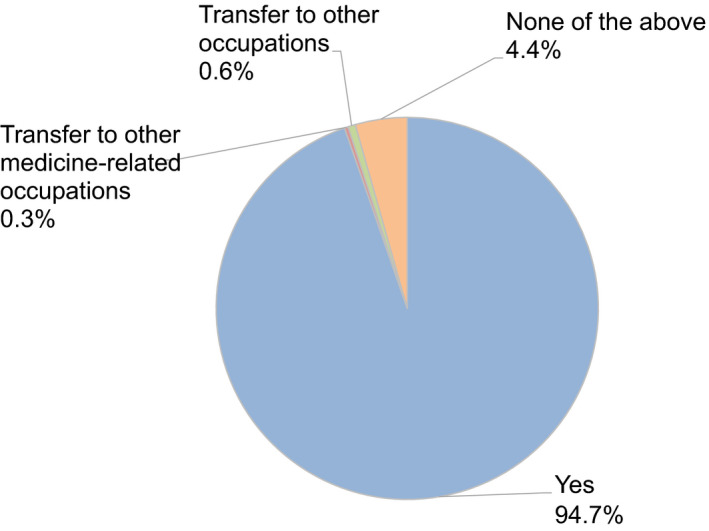
Reproductive medical providers’ intention to remain or change occupations after the end of the COVID‐19 pandemic

**FIGURE 12:**
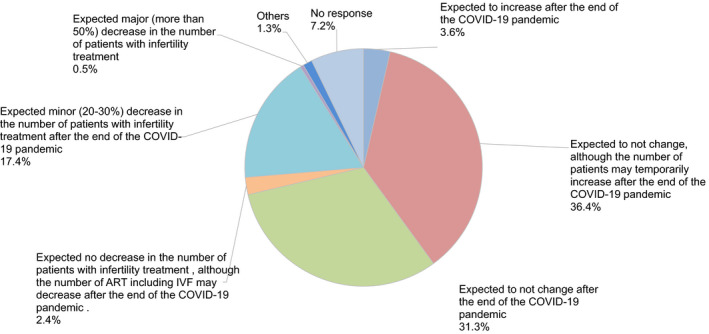
The future status of fertility treatment after the end of the COVID‐19 pandemic

Moreover, 40.4% and 19.3% of participants felt anxiety about their income and employment (Figure 10). Almost all participants (94.7%) hoped to continue engaging with couples undergoing fertility treatment (Figure 11), and 67.7% expected no change in the number of patients receiving fertility treatment, including ART, after the COVID‐19 pandemic ends. However, 17.4% of respondents think the number of patients with fertility treatment may decrease by 20 to 30% after the pandemic (Figure 12).

## 4 DISCUSSION

The current article presents a cross‐sectional overview of the impact of the COVID‐19 pandemic on the behaviors and responses of reproductive medical providers who work in a hospital or clinic for fertility treatments in Japan. During the COVID‐19 pandemic, almost all the participants indicated that they experienced a decrease in the number of patients and a decreased workload during the COVID‐19 pandemic.

In most European countries, ART activities were stopped in March, most often after a recommendation from the local authorities or national scientific society. As of the second half of April, treatments resumed gradually in different countries.^8^ In Italy, where the infection spread was particularly severe, fertility treatment was stopped completely for nearly two months.^9^ In Japan, where the peak of the COVID‐19 pandemic was different from that in most European countries, after the urgent declaration from the Japanese government and recommendations from the scientific society such as JSRM, this survey revealed that 73.2% of reproductive medical providers reported a decrease of outpatients undergoing fertility treatment and about 50% of participants are working less due to the COVID‐19 pandemic. Furthermore, many reproductive medical providers explained the risk of infection during pregnancy and did not postpone the fertility treatment but assured treatment when the patient wished to continue fertility treatment. Indeed, over half the couples have not halted the fertility treatment, including oocytes retrieval for IVF and all embryo freezing, although the epidemiologic curve hit the exponential phase (ie, when the daily increase of COVID‐19 patients was exponential) at that time and the Japanese government declared a state of emergency in response to the spread of COVID‐19. In contrast, in Europe, where the spread of infection and the peak of infection occurred earlier than in Japan, almost no patients received fertility treatment during late March and early April because of the forced or voluntary suspension or postponement of fertility treatment.^9^, ^10^ Thereafter, fertility treatment was resumed as soon as possible, and the period of discontinuation or postponement was only about seven weeks because the chance of infection and hence potential SARS‐CoV‐2‐related complications during pregnancy was reduced.^10^

A cessation of fertility treatments might impact the psychosocial health of infertile couples. This possibility was a factor to be considered, as mentioned in a statement from the American Society for Reproductive Medicine, ESRHE, and the International Federation of Fertility Societies.^9^ However, we might not have the answer as to whether fertility treatment needs to be suspended or postponed since there are a significant number of unknown factors concerning COVID‐19 and pregnancy following fertility treatment. Therefore, most data were extrapolated from previous experiences with other coronaviruses—SARS‐CoV and MERS‐CoV—as well as influenza pandemics, such as the 1918 flu and the Asian flu in the late 1950s.^11^, ^12^ There is limited evidence regarding SARS or MERS and pregnancy because SARS and MERS had limited spread; the rate of fatality for all reporting cases of SARS in pregnancy was higher as well as MERS in pregnancy.^13^, ^14^, ^15^, ^16^, ^17^ In addition, the miscarriage rate was 57.1% in women infected with SARS in very early pregnancy.^17^ A very recent report on COVID‐19 infection during pregnancy described that preterm delivery and preeclampsia are more common than in the general population, although clinical evidence of vertical transmission was importantly found in none of the newborns included. Additionally, there were no data on miscarriage for COVID‐19 infection occurring during the first trimester.^18^ In this survey, patients did not seem to be hesitant about becoming pregnant during the COVID‐19 pandemic, as only 2.8% of infertile couples postponed embryo transfer to avoid pregnancy, despite most health providers explaining the recommendation to cease fertility treatment stated by JSRM.

During this critical situation, health care workers on the front line who are directly involved in the diagnosis, treatment, and care of patients with COVID‐19 are at risk of developing psychological distress and other mental health symptoms. The ever‐increasing number of confirmed and suspected cases, overwhelming workload, depletion of personal protection equipment, widespread media coverage, lack of specific drugs, and feelings of being inadequately supported may all contribute to the mental burden of these health care workers. Previous studies have reported adverse psychological reactions to the 2003 SARS outbreak among health care workers.^4^, ^5^, ^6^, ^7^ Studies showed that those health care workers feared contagion and infection of their family, friends, and colleagues,^4^ felt uncertainty and stigmatization,^4^, ^5^ reported reluctance to work or contemplating resignation,^6^ and reported experiencing high levels of stress, anxiety, and depression symptoms,^7^ which could have long‐term psychological implications.^7^ Similar concerns about the mental health, psychological adjustment, and recovery of health care workers treating and caring for patients with COVID‐19 are now arising. Indeed, some studies reported that reproductive medical providers who frequently contact patients reported high rates of symptoms of depression, anxiety, insomnia, and distress during the COVID‐19 epidemic.^19^, ^20^, ^21^, ^22^, ^23^ Xiang et al ^24^ mentioned that based on experience from past severe global outbreaks of novel viruses like SARS and the psychosocial impact of viral epidemics, the implementation of mental health assessment, support, treatment, and services are crucial and pressing goals for sustaining health and improvement of reproductive medical providers to the COVID‐19 outbreak.^25^

Of note, the majority of the participants in this survey felt that most reproductive medical providers have a fear of infection with COVID‐19 in patient‐facing clinical settings. One of the reasons for this is that more than 40% of the participants in this survey reported a lack of personal protection equipment (data not shown), which may explain, in part, their fear of infection caused by lack of supplies. Therefore, effective strategies toward improving mental health established by the comprehensive psychological consultation organization and hospital‐developed detailed rules on the use and management of protective equipment should be provided to these individuals to reduce worry. In contrast, most of the participants were eager to continue working.

About 90% of the health providers answered “no” or “neither” to the question of whether fertility treatment is necessary during the COVID‐19 pandemic (only 11% answered “yes”). So far, it is difficult for reproductive medical providers to say whether fertility treatments are currently necessary (advisable) or not because of limited evidence. Hence, we may need further studies with long‐term follow‐ups of children born following fertility treatments in Japan.

The NIH in the US has already begun the Assessing the Safety of Pregnancy in the Coronavirus Pandemic (ASPIRE) Study, a prospective nationwide cohort study of pregnant women enrolled early in gestation and followed for COVID‐19 exposure and infection, with follow‐up of obstetrical outcomes and infant development through the first year of life. Nevertheless, some specific sectors of the population, such as women with advanced maternal age and/or diminished ovarian reserve, definitively agreed to proceed with treatment and fertility preservation for oncological patients so as not to lose precious time to enhance the pregnancy opportunities of these women. Therefore, many reproductive medical providers engaged in reproductive medicine, including ART, needed to consider a major reassignment of medical support for patients since scientific evidence is lacking about the effect of COVID‐19 infections during pregnancy. Furthermore, not only the reproductive medical provider but also the patients face confusion regarding whether reproductive medicine, including all interventions such as ART, IUI, and natural course, is necessary or not during the COVID‐19 pandemic, although nobody has the answer whether to continue reproductive medicine contrary to some social recommendations.

This study has several limitations. First, this survey was carried out for six days, and it seems that activity was restarted as soon as a decline in the curve of daily new confirmed cases was established. Additionally, this study lacks longitudinal follow‐up. Going ahead, behaviors and responses, including the mental health symptoms of reproductive medical providers, could change. Thus, the long‐term implications of this population are worthy of further investigation. Second, the responses to this survey may vary as the impact of the COVID‐19 epidemic differs between urban and rural areas. Therefore, if the survey had been conducted only in areas where the infection had spread, such as Tokyo, the results could have been very different. Since this survey was conducted with reproductive medical providers, results may differ from those using similar questionnaires for patients. However, there were also some strengths in this study. We investigated a very important and current medical issue using a large sample of health professionals, and we were able to recruit them in a very short time. This rapid and easy‐to‐use web‐based methodology may be useful for further investigations because of the ease with which researchers can reach out to additional targets.

In this survey, documented data such as those presented in this article will provide a basis for further study regarding behaviors and responses of reproductive medical providers to the COVID‐19 crisis. Such conclusions will be valuable for health authorities and health care professionals in case of a future global pandemic. This paper reflects the current state of reproductive health care at a time when we have been forced into a new way of life by an emerging global infectious disease that we have never experienced before. The findings may form the basis for improving the current situation and can be used in the future when similar crises arise.

## CONFLICT OF INTEREST

Kuniaki Ota, Osamu Tsutsumi, Tasuku Mitani, Yoshiharu Morimoto, Atsushi Tanaka, Yutaka Osuga, Toshifumi Takahashi, and Yoshihiko Hosoi declare that they have no conflict of interest. *Human rights statements and informed consent*: All procedures followed were in accordance with the ethical standards of the responsible committee on human experimentation (institutional and national) and with the Helsinki Declaration of 1964 and its later amendments. Informed consent was obtained from all participants included in the study. The Institutional Review Board of HORAC Grand Front Osaka Clinic approved this study (Number:2020‐31). *Animal rights*: This report does not contain any studies performed by any of the authors that included animal participants.
